# A lipid metabolism-related genes prognosis biomarker associated with the tumor immune microenvironment in colorectal carcinoma

**DOI:** 10.1186/s12885-021-08902-5

**Published:** 2021-11-05

**Authors:** Chao Yang, Shuoyang Huang, Fengyu Cao, Yongbin Zheng

**Affiliations:** Department of Gastrointestinal Surgery, Renmin Hospital of Wuhan University, No. 99 ZhangZhiDong Street Wuchang District, Wuhan, 430060 Hubei Province People’s Republic of China

**Keywords:** Colorectal cancer, Lipid metabolism-related genes, Prognostic value, Tumor immune microenvironment

## Abstract

**Background and aim:**

Lipid metabolic reprogramming is considered to be a new hallmark of malignant tumors. The purpose of this study was to explore the expression profiles of lipid metabolism-related genes (LMRG) in colorectal cancer (CRC).

**Methods:**

The lipid metabolism statuses of 500 CRC patients from the Cancer Genome Atlas (TCGA) and 523 from the Gene Expression Omnibus (GEO GSE39582) database were analyzed. The risk signature was constructed by univariate Cox regression and least absolute shrinkage and selection operator (LASSO) Cox regression.

**Results:**

A novel four-LMRG signature (PROCA1, CCKBR, CPT2, and FDFT1) was constructed to predict clinical outcomes in CRC patients. The risk signature was shown to be an independent prognostic factor for CRC and was associated with tumour malignancy. Principal components analysis demonstrated that the risk signature could distinguish between low- and high-risk patients. There were significantly differences in abundances of tumor-infiltrating immune cells and mutational landscape between the two risk groups. Patients in the low-risk group were more likely to have higher tumor mutational burden, stem cell characteristics, and higher PD-L1 expression levels. Furthermore, a genomic-clinicopathologic nomogram was established and shown to be a more effective risk stratification tool than any clinical parameter alone.

**Conclusions:**

This study demonstrated the prognostic value of LMRG and showed that they may be partially involved in the suppressive immune microenvironment formation.

**Supplementary Information:**

The online version contains supplementary material available at 10.1186/s12885-021-08902-5.

## Background

Colorectal cancer (CRC) has the third highest incidence among malignancies worldwide and is the second most common cause of cancer-related deaths [1]. As the most common gastrointestinal malignancy, CRC is associated with well-known risk factors, including poor dietary patterns, obesity, alcoholic abuse, smoking, and physical inactivity. Besides environmental influences, divergent genomic factors also contribute to the complexity of CRC [2]. Despite clinical application of cancer screening procedures and effective treatments, the morbidity and mortality of CRC remain consistently elevated in most countries, especially in economically underdeveloped regions [3]. Conventional therapeutic approaches, such as surgical resection combined with chemotherapy, have been shown to improve survival and quality of life; however, the overall survival (OS) of CRC patients remains unsatisfactory [4]. Although the 5-year survival rate of patients with early CRC (stage I and II disease) is more than 60%, it is less than 10% for stage IV patients and some stage III patients with metachronous distant metastasis [3, 5]. Hence, a reliable risk prediction model is essential to improve the clinical prognosis of CRC by providing targeted screening and individualized intervention measures [6]. At present, the tumor node metastasis (TNM) staging is still the most widely used method for prognostic evaluation in CRC [7]. Other predictive factors include pathological type, differentiation degree and microvascular/serosa invasion, etc. However, these clinicopathological biomarkers cannot provide precise prognostic guidelines as they do not fully capture disease information. With advances in next-generation sequencing, inter- and intra-tumor molecular heterogeneity has been highlighted, increasing the difficulty of risk stratification [8]. Therefore, molecular characteristics of tumors need to be included in new prognostic risk models.

Accumulating evidence indicates that tumor metabolic heterogeneity is related to clinical outcome, epigenetics status, and treatment resistance [9–11]. Oncogene-driven metabolic reprogramming improves cellular fitness to meet bioenergetic, biosynthetic, and redox balance demands, thereby providing a selective advantage during tumorigenesis [12]. Metabolic reprogramming is considered to be a new hallmark of malignant tumors [13]. Although most studies of alterations in cancer metabolism have focused on glucose metabolism (i.e., the Warburg effect), the role of abnormal lipid metabolism in cancer cells has been gradually recognized over the past few years [14, 15]. A rapidly proliferating cancer cell requires more energy than a normal cell and meets its biological needs by activating an endogenous production pathway or increasing its intake [16]. ATP generated by fatty acid oxidation is an important energy source for cancer cells when energy provision is insufficient. Adipocytes and free fatty acids in the hypoxic tumor microenvironment are markedly conducive to cancer proliferation, progression, invasion, and metastasis [17, 18]. It has been reported that cancer cells rely mostly on endogenous adipogenesis rather than uptake of exogenous fatty acids, which is more common in normal cells [19]. Hence, abnormal lipid metabolism, especially fatty acid synthesis and oxidation, is increasingly regarded as an important feature of metabolic reprogramming.

Epidemiological studies have shown that serum triglyceride levels are associated with susceptibility to CRC [20, 21]. Wang et al. confirmed that alterations in the abundances of individual lipids were present in CRC using shotgun lipidomics [22]. The study showed increased expression of lipogenic enzymes involved in de novo adipogenesis (fatty acid synthesis) in CRC, including fatty-acid synthase, acetyl-CoA carboxylase, and carnitine palmitoyltransferase, whereas enzymes involved in fatty acid oxidation showed decreased expression. As a mitochondrial serine/threonine phosphatase, PGAM5 regulates a variety of metabolic pathways in vivo. Research by Zhu et al. showed that blocking PGAM5 would reduce lipid metabolism and inhibit the occurrence of CRC in mice [23]. Gong and his colleagues showed that metabolism reprogramming in tumor-associated fibroblasts significantly enhanced the invasion and metastasis of CRC [24]. Besides, drug resistance to antiangiogenic therapy frequently arises during cancer treatment; the underlying molecular mechanism of this resistance might include lipid metabolism reprogramming [25]. Iwamoto et al. observed that blocking carnitine palmitoyl transferase 1A (CPT1A), a key enzyme in lipid metabolism, could restore sensitivity to antiangiogenic therapy [18]. Thus, they introduced a promising approach to overcoming drug resistance to cancer therapies by combining conventional therapy and targeted lipid metabolism.

Previous studies have confirmed the close relationship between altered lipid metabolism and CRC in tumorigenesis, progression and treatment. A signature of lipid metabolism-related genes (LMRG) was shown to have high prognostic value in papillary thyroid cancer and diffuse glioma [26, 27]. In-depth study of lipid metabolomics characteristics could lead to better understanding of the progression of tumors and provide potential metabolic targets for the development of new treatment methods [28]. However, the prognostic value of LMRG in CRC has not been verified by large-sample studies. The present study aimed to develop a novel risk signature based on LMRG to provide additional information for use in risk assessment and clinical decision-making in CRC.

## Methods

### Data collection

**Fig. 1:**
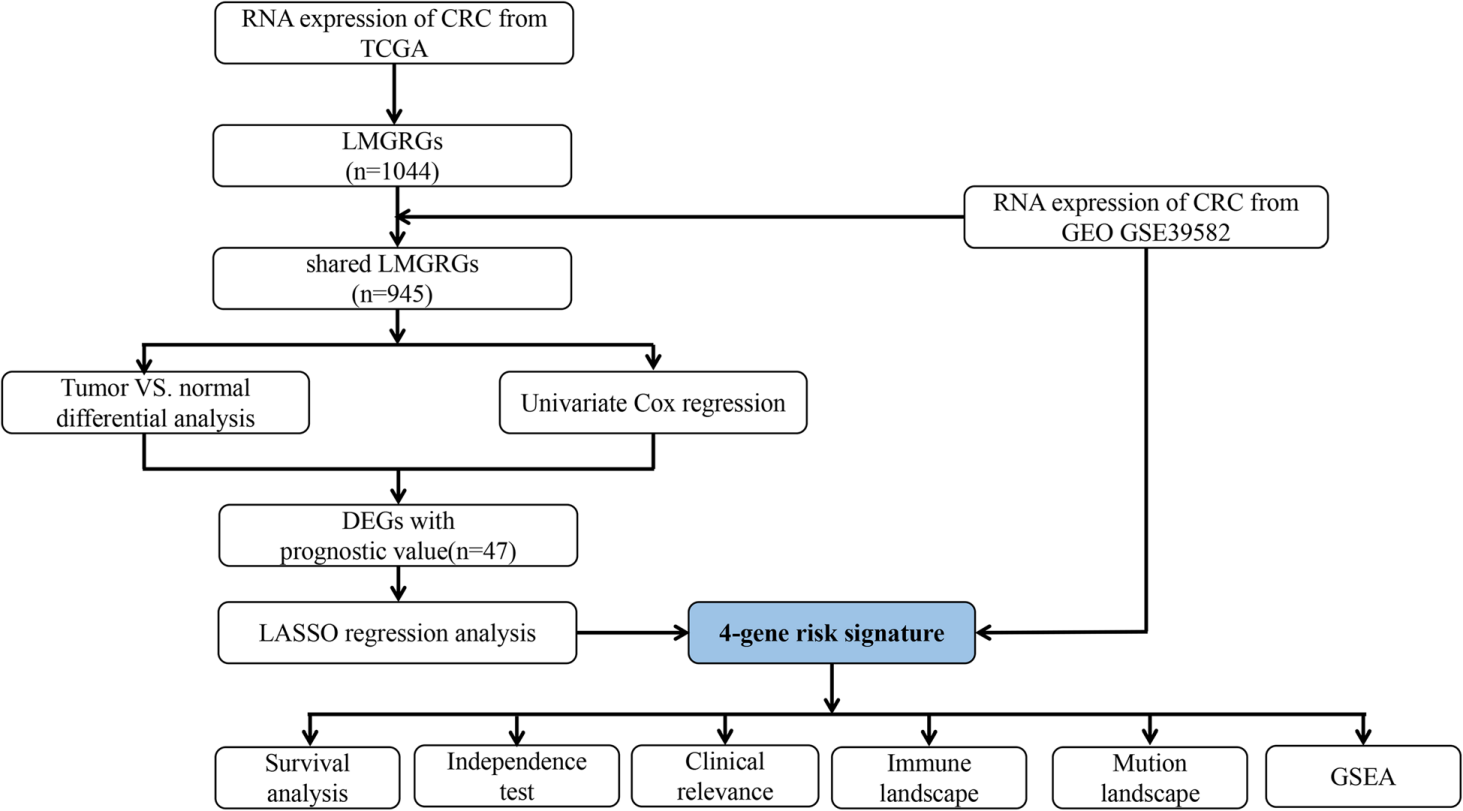
Flowchart of study design

A flow chart of this research is presented in Fig. 1. Level 3 RNA sequencing data (RNA-seq) data, mutation data, and matched clinical information were obtained from the TCGA CRC cohort (Data Release 25.0 - July 22, 2020, *https://portal.gdc.cancer.gov/repository*). The data search and selection strategy were as follows. (1) The keywords for cases were “colon and rectum [Primary Site]”, “TCGA [Program]”, “TCGA-COAD, TCGA-READ [Project]”, “Adenomas and Adenocarcinomas [Disease Type]”. (2) The keywords for files were “Transcriptome Profiling [Data Category]”, “Gene Expression Quantification [Data Category]”, “RNA-Seq [Experimental Strategy]”, “HTSeq-FPKM [Workflow Type]”, “.txt Format [Data Format]” . The RNA-seq matrix file was annotated using the human General Transfer Format (hunman.gtf) from the Ensembl database (https://www.ensembl.org/) with the Strawberry Perl software (version 5.28.2.1, https://strawberryperl.com/). The RNA-seq transcriptome data (FPKM) from the TCGA cohort were converted to log_2_(TPM + 1) to obtain normalized counts.

Raw CEL and clinical data for CRC were downloaded from the Gene Expression Omnibus (GEO) (*https://www.ncbi.nlm.nih.gov/geo/*); the search used the keywords “colon cancer”, “rectum cancer”, “adenocarcinoma”, “*Homo sapiens*”, and “Expression profiling.” The gene expression microarray dataset GSE39582 was selected and downloaded because it had the largest sample size for CRC [29]. Probe IDs were matched to gene symbols using the GPL570 platform (Affymetrix Human Genome U133 Plus 2.0 Array). The mean expression value of the probes was used as the expression value for the gene in question if multiple probes were mapped to a single gene. The robust multichip average (RMA) was used to normalize the raw data from GEO cohort by the R package *affy* [30].

Criteria for study exclusion were: (1) patients with unknown survival status, follow-up information, and disease stage; and (2) patients who died within a follow-up period of 30 days. Consequently, 544 cases (500 tumor and 44 normal samples) meeting the criteria were included in the training set, and 542 cases (523 tumor and 19 normal samples) were included in the validation set (Supplementary Table 1). The TCGA cohort was used to establish a risk signature, and the GEO set was used for validation. Both the TCGA and GEO databases are publicly available; therefore, the present study did not require approval from the local ethics committees.

### Identification of LMRG

The five LMRG sets were collected from the Molecular Signature Database [27, 31] (Supplementary Table 2). A total of 1044 genes were found to be involved in the lipid metabolism process after removal of overlapping genes.

### Construction of risk signature based on LMRG

The shared LMRG in the GEO and TCGA sets were selected for subsequent analysis. Differentially expressed genes (DEGs) between tumor and normal samples were screened in the TCGA cohort using the R package *limma* and *sva* with a false discovery rate (FDR) < 0.05 [32, 33]. Gene ontology (GO) and KEGG analyses of DEGs were performed using R package *clusterProfiler, org. Hs.eg.db, enrichplot* and *ggplot2* [34, 35]. The LMRG with prognostic value was identified using univariate Cox analysis. Next, the overlapping genes between DEGs and prognostic-related genes were identified using a Venn diagram for subsequent analysis (http://bioinformatics.psb.ugent.be/webtools/Venn/). Least absolute shrinkage and selection operator (LASSO) Cox regression was used to select the best prediction model based on these mutual genes in TCGA CRC patients [36]. LASSO analysis was performed using R package *glmnet* and *survival*, and the optimal value of the penalty parameter was determined by 10-fold cross-validation [37]. Risk signatures were generated from the TCGA and GEO cohorts based on the expression levels of LMRG and the corresponding coefficients. The risk score of each sample was calculated by the following formula: (expGene: the expression level of LMRG in the TCGA or GEO cohort; Coef: the coefficient of LMRG in the LASSO Cox regression model in the training set).

$$ risk\kern0.17em score=\sum \limits_{i=1}^n\left(\exp Genei\times Coefi\right) $$

### Prognostic value of the risk signature in training and validation group

The patients were stratified into high- and low-risk groups according to the median value of the risk score. Kaplan Meier (K-M) survival curves with the Log-rank test were constructed to demonstrate the prognostic ability of the risk signature. In addition, the area under the curve values (AUCs) of the receiver operating characteristic (ROC) curves for 1-, 3-, and 5-year survival were calculated using R package *survivalROC* to evaluate the performance of those two signatures [38].

### Gene set enrichment analysis (GSEA)

To explore potential molecular mechanism between the two groups, GSEA was carried out between the high- and low-risk groups. The annotated gene set list, h.all.v7.2.symbols.gmt (Hallmarks), was selected as the reference gene set from the Molecular Signature Database [31].

### Independence of the risk signature from other clinicopathological parameters

To determine the independence of the risk signature from other clinical parameters, univariate and multivariate analyses of the risk score with age, gender, and tumor stage were performed. Forest plots were used to show the independence of the risk score.

### Correlation between the risk signature and other clinicopathological parameters

The associations between the risk signature and clinicopathological parameters in TCGA -CRC cohort, including age, gender, tumor stage, pathological T stage, N stage, and M stage, were further explored. Patients were stratified into subgroups of age ≤ 65 years and age ≥ 65 years, female and male, pathological tumor stage I + II and stage III + IV, T1 + T2 and T3 + 4, N0 and N1 + 2, M0 and M1. K-M survival analysis of the aforementioned paired subgroups was performed.

Cancer stem cells are highly dependent on lipid metabolism to maintain their stem cell characteristics. One study showed that cancer stem cells in CRC have higher lipid metabolism levels than tumor cells or normal colonic epithelial cells [39]. Malta et al. had developed a novel analysis tool to assess stemness features based on gene expression [40]. In this study, the mRNA expression-based stemness index (mRNAsi) for each CRC patient was obtained from the results of Malta et al. Besides, CD133 is a marker gene of many tumor stem cells. Hence, the relationships of the risk score with the mRNAsi score and CD133 mRNA were also analyzed.

### Nomogram construction and validation

A nomogram integrating the risk signature and other clinicopathological factors was established for prognostic evaluation using the R package *rms* (*https://CRAN.R-project.org/package=rms*). The AUCs of the ROC curve were demonstrated to assess the predictive capability of the nomogram. Calibration curves were also established to evaluate the predictive accuracy of the nomogram.

### Estimation of relative abundance of immune cell types in different risk groups

The CIBERSORT algorithm *(**https://cibersort.stanford.edu**)*, which quantifies the relative abundance of immune cells based on specific gene expression profiles, is used to assess the distribution of 22 immune cell types in CRC samples from TCGA cohort [41]. The *P*-value, correlation coefficient, and root mean squared error were also calculated to evaluate the accuracy of the results for each patient. Samples with *P* < 0.05 were used to compare immune cell abundance in different risk groups.

### Mutation analysis

The top 20 genes with the highest mutation frequency in TCGA -CRC cohort were analyzed in both high- and low-risk groups using the R package *GenVisR* [42]. Visualization based on the somatic mutation data in Mutation Annotation was performed using the R package *maftoools* [43]. Tumor mutational burden (TMB) is known to be associated with the efficacy of adjuvant chemotherapy (fluoropyrimidine plus oxaliplatin regimen) in CRC [44, 45]. TMB is also an independent predictor of immunotherapy response in a variety of tumors [38, 39]. Therefore, the relationship between risk score and TMB was also explored in this study. The TMB score was generated as the total number of somatic mutations divided by the number of exons in each sample [46]. The exon size is often approximately estimated at 38 megabases.

### Statistical analysis

All the statistical analyses and drawings in this study used R (version 3.6.3) or GraphPad Prism (version 8.3.0). Differences between continuous variable were analyzed using *t*-test. Fisher’s exact test or chi-square test was employed for comparisons of categorical variables. Log-rank test was used to estimate the differences among K-M survival curves. *P* < 0.05 (two-tailed) was considered significant.

## Results

### Identification of differentially expressed and prognosis-related genes in LMRG

**Fig. 2:**
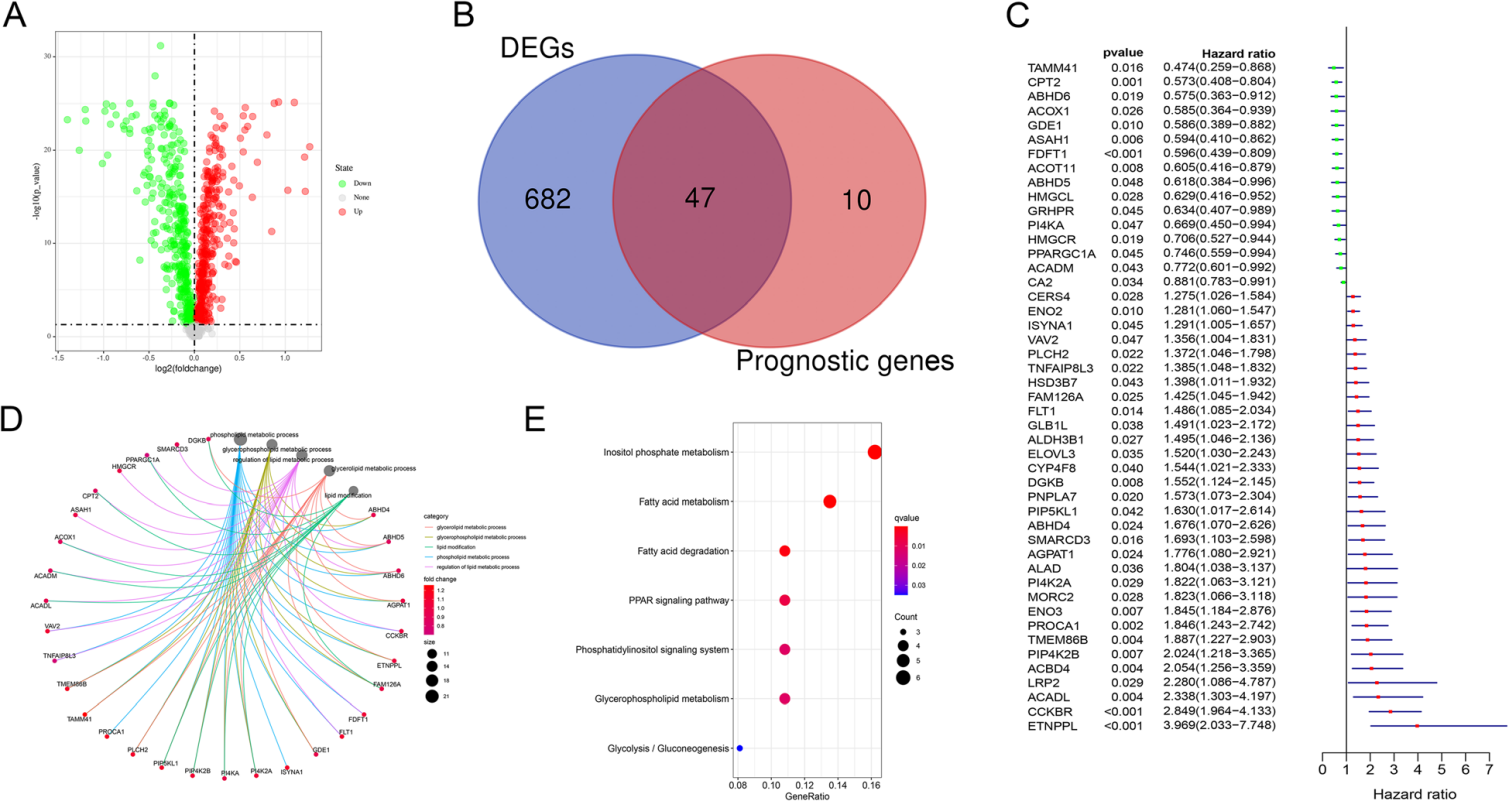
Identification of the differentially expressed and prognosis-related genes in the TCGA cohort. (**A**) Volcano map showing the differentially expressed genes between normal and tumor tissues. The X axis indicates the -log(*P*-value), and the Y axis indicates the log_2_(fold change). Red dots represent upregulated genes; green dots represent downregulated genes. (**B**) Venn diagram showing the 47 DEGs that also had prognostic value. The left half of the Venn diagram shows the differentially expressed genes between normal and tumor tissues. The right half of the diagram shows the prognosis-related genes. (**C**) Univariate Cox regression analysis of the 47 overlapping genes. (**D-E**) The gene ontology (GO) enrichment terms (**D**) and KEGG pathways (**E**) of the 47 overlapping genes were both concentrated mainly in lipid metabolic processes

Expression data of LMRG were extracted from the TCGA and GEO cohorts. A total of 945 shared LMRG were matched. There were 729 DEGs between normal and tumor tissues, including 365 upregulated and 364 downregulated genes, in the training cohort when the cut-off was set to FDR < 0.05 (Fig. 2A). Univariate Cox analysis was employed to filter genes with prognostic value from the 945 intersecting genes. Finally, 57 LMRG were shown to be related to prognosis in the training set. A total of 47 shared LMRG were found to be both DEGs and prognostic genes according to the Venn diagram (Fig. 2B, C).

Functional enrichment analysis identified terms related to lipid metabolism. The top five biological process terms were glycerolipid metabolic process, glycerophospholipid metabolic process, lipid modification, phospholipid metabolic process, and regulation of lipid metabolic process (Fig. 2D). Kyoto Encyclopedia of Genes and Genomes (KEGG) analysis showed that the 47 shared LMRG were mainly involved in glycerophospholipid metabolism, phosphatidylinositol signaling system, PPAR signaling pathway, fatty acid degradation, and fatty acid metabolism (Fig. 2E).

### Construction and validation of gene signature

**Fig. 3:**
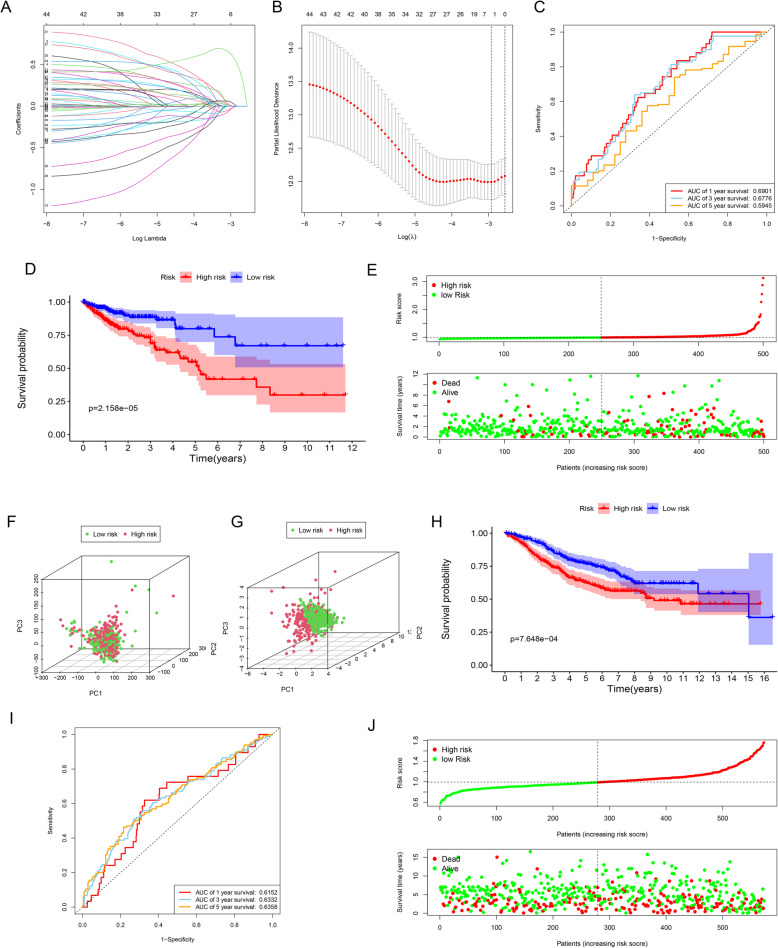
Development and validation of the risk signature. (**A**) LASSO Cox regression coefficient profiles of the 47 LMRG. Each curve represents the changing trajectory of one LMRG. (**B**) 10-fold cross-validation for tuning parameter selection in LASSO model. Each red dot represents a lambda value with a confidence interval. The two dotted lines indicate the optimal values by minimum criteria and 1-SE criteria by 10-fold cross-validation (standard error; SE). The Y-axis shows partial likelihood deviance values with error bars, and the X-axis shows the penalization coefficient (logλ). (**C**) Time-dependent ROC curves for 1-, 3-, and 5-year OS in the training group. (**D**) The survival curves showed significant differences between the high- and low-risk groups (*P* < 0.001). (**E**) Risk score distribution (above) and survival status (below) of CRC patients in different risk groups. (**F**) PCA showed no significant difference in risk status of CRC patients on the basis of the whole gene set. Different color points represent patients with different risk groups. (**G**) PCA showed that the high-risk group could be distinguished effectively from the low-risk groups based on the risk signature. (**H**) The survival curves showed significant differences between high- and low-risk patients in the validation set (*P* < 0.001). (**I**) Time-dependent ROC curves for 1-, 3-, and 5-year OS in the validation set. (**J**) Risk score distribution (above) and survival status (below) of CRC patients in the validation set

Next, the expression profiles of the 47 LMRG were used to establish a risk signature using LASSO Cox regression analysis (Fig. 3A, B). Finally, four LMRG, namely PROCA1, CCKBR, CPT2, and FDFT1, were used to establish the optimal lipid metabolism-related risk signature. The risk score for each patient was calculated by the following formula: risk score = (PROCA1*0.03071) + (CCKBR*0.58956) + (CPT2*0.00972) + (FDFT1*0.01381).

The predictive value of this risk signature was evaluated using ROC curves. The AUCs of the signature were 0.6901 at 1 year, 0.6776 at 3 years, and 0.5945 at 5 years in the training set (Fig. 3C). The patients were dichotomized into two risk group according to the median risk score. K-M survival curves showed that high-risk patients had significantly shorter OS than low-risk cases in the training set (Fig. 3D, *P* < 0.001). As shown in Fig. 3E, more patients died in the high-risk group, whereas the majority survived in the low-risk group. In the training set, principal components analysis (PCA) was used to obtain expression patterns in the low- and high-risk groups. There was no significant difference in risk status between the two groups when PCA was performed with all genes (Fig. 3F). However, the risk groups could be distinguished using the risk signature (Fig. 3G).

In the validation set, the high-risk patients again had significantly shorter OS than those in the low-risk group (Fig. 3H, *P* < 0.05). The AUCs were 0.6152 at 1 year, 0.6332 at 3 years, and 0.6358 at 5 years (Fig. 3I). The risk score distribution curve and survival status are shown in Fig. 3J.

### Independent prognostic value of the risk signature

**Fig. 4:**
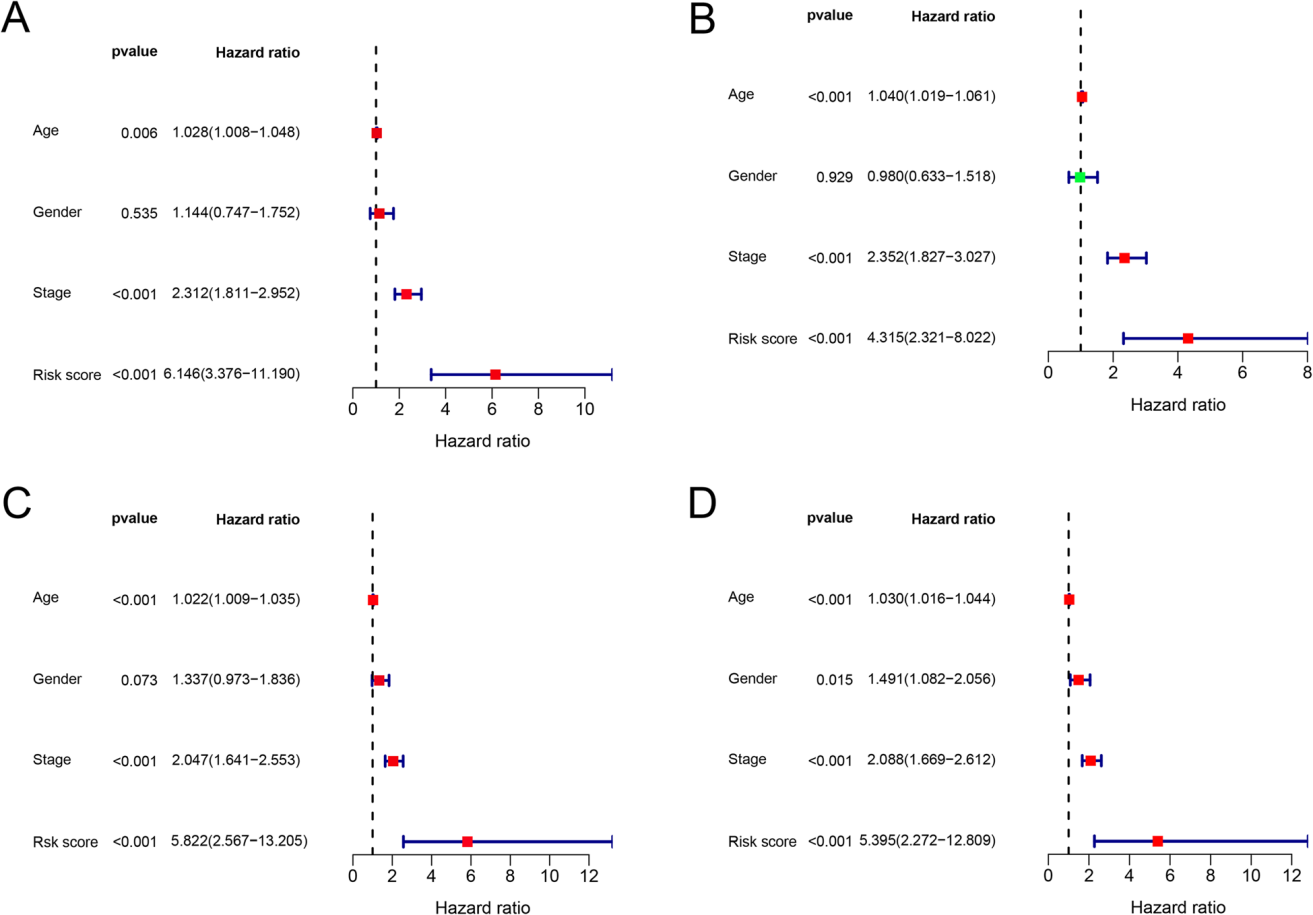
Univariate and multivariate Cox regression analysis showed that the risk score was an independent prognostic factor both in the training and validation sets. (**A**) Results of univariate Cox regression analysis in the training set. (**B**) Results of multivariate Cox regression analysis in the training set. (**C**) Results of univariate Cox regression analysis in the validation set. (**D**) Results of multivariate Cox regression analysis in the validation set

The results showed that the risk score based on the four-LMRG signature was an independent prognostic factor in the training set, with hazard ratio (HR) = 6.146 (95% confidence interval (CI) = 3.376–11.190 (*P* < 0.001; Fig. 4A) by univariate Cox analysis, and HR = 4.315 (95% CI = 2.321–8.022; *P* < 0.001; Fig. 4B) by multivariate Cox analysis. Similar results were obtained in the validation set, showing the independence of the risk signature with HR = 5.822 (95% CI = 2.567–13.205; *P* < 0.001; Fig. 4C) and HR = 5.395 (95% CI = 2.272–12.809; *P* < 0.001; Fig. 4D) by univariate and multivariate Cox analysis, respectively.

### Relationships between risk score and clinicopathological features

**Fig. 5:**
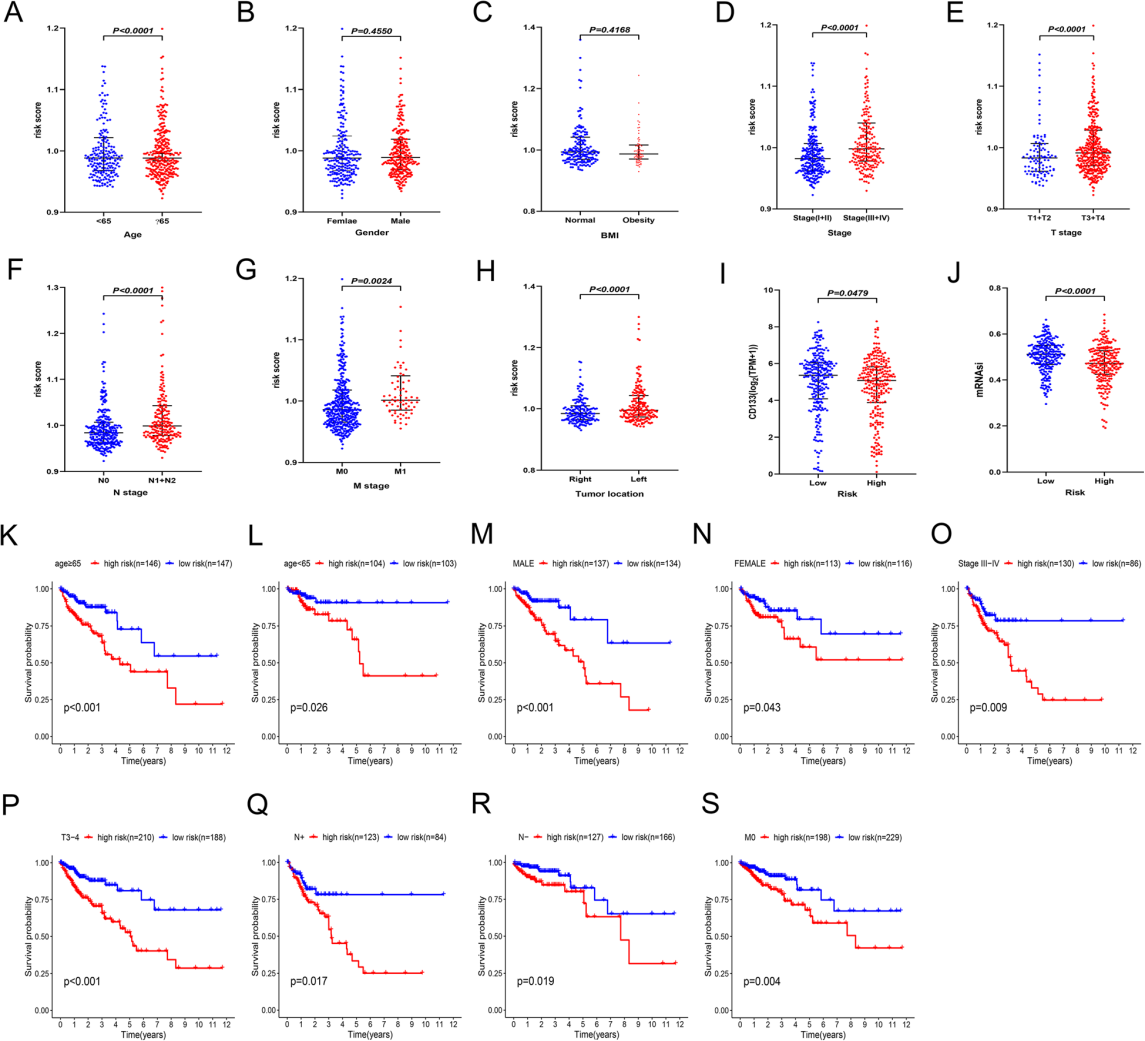
Stratified analysis of the risk signature. (**A**-**J**) Relationships between the risk signature and age (**A**), gender (**B**), BMI (**C**), tumor stage (**D**), T stage (**E**), N stage (**F**), M stage (**G**), tumor location (**H**), CD133 mRNA (**I**), and mRNAsi (**J**). (**K**-**S**) Poor prognosis was observed in the high-risk group in the majority of clinical stratifications, including age (**K** and **L**), gender (**M** and **N**), tumor stage (**O**), T stage (**P**), N stage (**Q** and **R**) and M stage (**S**)

Risk score was significantly associated with age (*P* < 0.0001; Fig. 5A) but not gender (*P* = 0.4168; Fig. 5B). In addition, the relationship between obesity and risk score was explored. Obesity was defined as a body mass index (BMI) equal to or greater than 30 [47]. Interestingly, no significant difference in lipid metabolism-related risk score was found between the normal weight and obese groups (*P* = 0.4168; Fig. 5C).

The results showed that risk score was positively correlated with the degree of tumor progression. For instance, the risk scores in TNM stage III + IV patients were markedly higher than those in tumor stage I + II (*P* < 0.0001; Fig. 5D). Similar results were obtained in other subgroups, including pathological T stage (I + II vs. III + IV, *P* < 0.0001; Fig. 5E), N stage (N0 vs. N1 + 2, *P* < 0.0001; Fig. 5F), M stage (M0 vs. M1, *P* = 0.0024; Fig. 5G), and tumor location (left vs. right, *P* < 0.0001; Fig. 5H). There were also more stem cell characteristics in the low-risk group, including significantly higher levels of CD133 mRNA (*P* = 0.0479; Fig. 5I) and mRNAsi (*P* < 0.0001; Fig. 5J).

Subgroup analysis was performed to further assess whether the risk signature still had independent prognostic value within specific clinical parameters. The results showed that the risk signature retained its powerful prognostic prediction ability in subgroups based on age (age ≥ 65 or < 65, Fig. 5K and L), gender (male or female, Fig. 5M or 5N), tumor stage (III + IV, Fig. 5O), T stage (III + IV, Fig. 5P), N stage (N1 + 2 or N0, Fig. 5Q and R), M stage (M0, Fig. 5S). The OS of high-risk patients was significantly shorter than that of low-risk patients in the different clinicopathological characteristic subgroups.

### Construction and validation of a nomogram combining clinicopathological features and risk signature

**Fig. 6:**
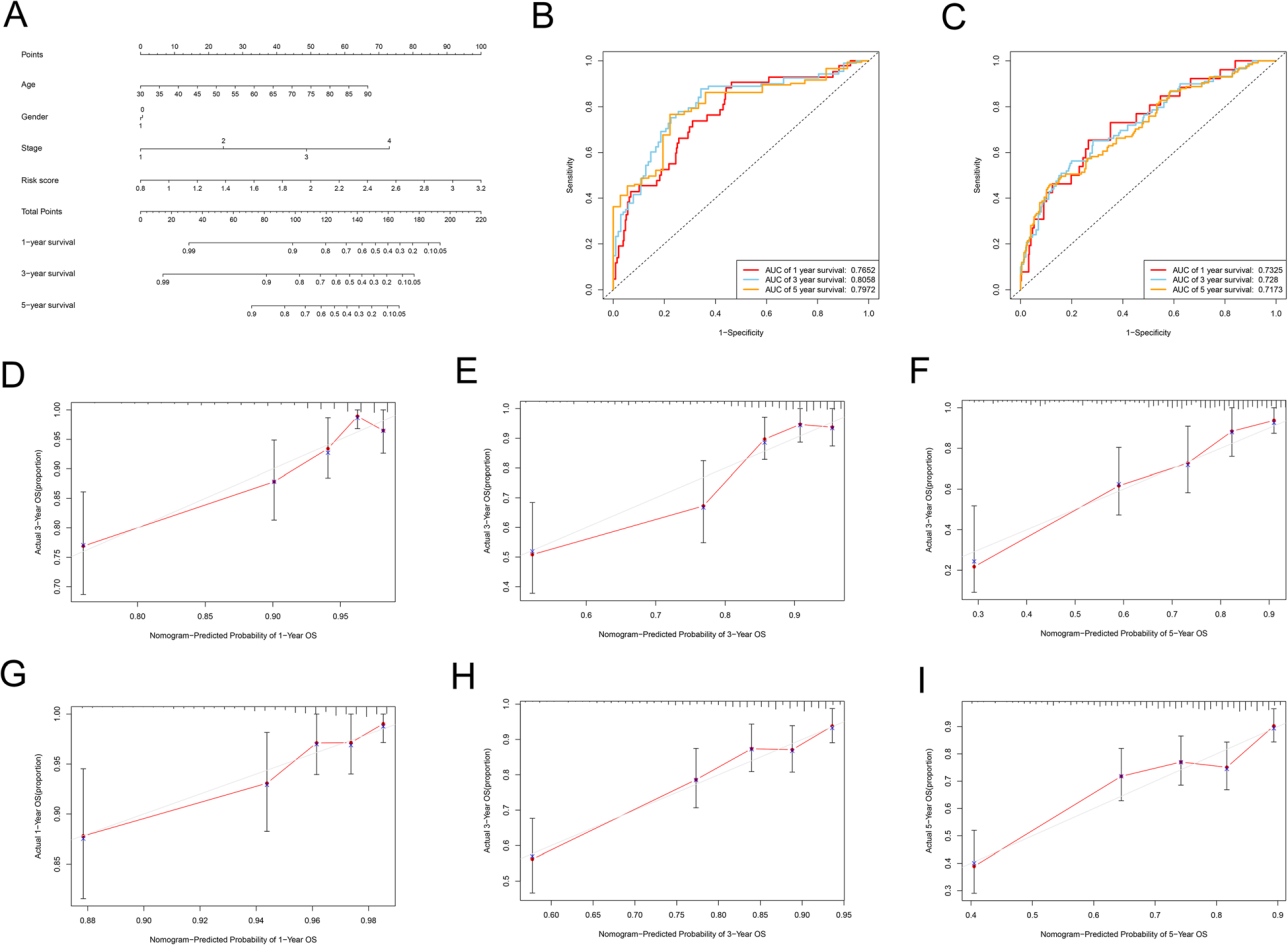
A genomic-clinicopathologic nomogram for prognostic prediction in CRC. (**A**) The nomogram included four variables (age, gender, disease stage, and risk score). Each variable in the nomogram was assigned a weighted score based on the multivariate Cox regression coefficient. To use this nomogram, the specific value of an individual variable is located on each axis, and a line is drawn upward to the Points axis to obtain the score of this variable. In the same way, the scores of four variables are summed to give the total score. The total score is located on the Total Points axis, and a line is drawn downwards to the survival axes to determine the 1-, 3-, and 5-year survival probability. (**B**) Prognostic value of the nomogram for predicting 1-, 3-, and 5-year overall survival rate in the training set. (**C**) Prognostic value of the nomogram for predicting 1-, 3-, and 5-year OS rates in the validation set. (**D**-**F**) Calibration plots suggest that the nomogram’s predictions of 1-year (**D**), 3-year survival (**E**), and 5-year survival (**F**) match well with the actual observed probabilities in the training set. The actual survival rate and nomogram-predicted probabilities were plotted on the vertical and horizontal axes, respectively. Dashed line at 45° represents perfect prediction and the actual performances of our nomogram are red line. The more the blue lines and dashed lines in the graph coincide, the better the predictive performance of the nomogram. The calibration curve of the nomogram is mainly assessed by observing the degree of consistency between the predicted curve and the ideal curve in the graph. (**G**-**I**) The calibration plots showed that the actual observed probabilities were in agreement with the predictive values from the nomogram for 1-year (**G**), 3-year (**H**), and 5-year survival (**I**) in the validation set

A nomogram was constructed based on several factors, namely age, gender, disease stage, and risk score, to provide a visualization tool for clinicians to predict the probability of 1-, 3- and 5-year OS in CRC patients. A total score could be calculated for each patient using this nomogram, where a higher total score indicates a worse outcome (Fig. 6A). This nomogram had high potential for clinical utility, with ROC AUC values of 0.7652 at 1 year, 0.8058 at 3 years, and 0.7972 at 5 years in the training set (Fig. 6B). Moreover, the calibration plots indicated that the actual observation probability was very close to the predictive probability of the nomogram (Fig. 6D-F).

The nomogram for the validation set is shown in Fig. 6D. The AUCs of the ROC curve were 0.7325 at 1 year, 0.7280 at 3 years, and 0.7173 at 5 years (Fig. 6C). The predictive probabilities obtained using the nomogram showed good consistency with the actual observed values (Fig. 6G-I). Hence, the prognostic nomogram model was considered to be robust.

### Immune landscapes differ among two risk subgroups in TCGA -CRC cohort

**Fig. 7:**
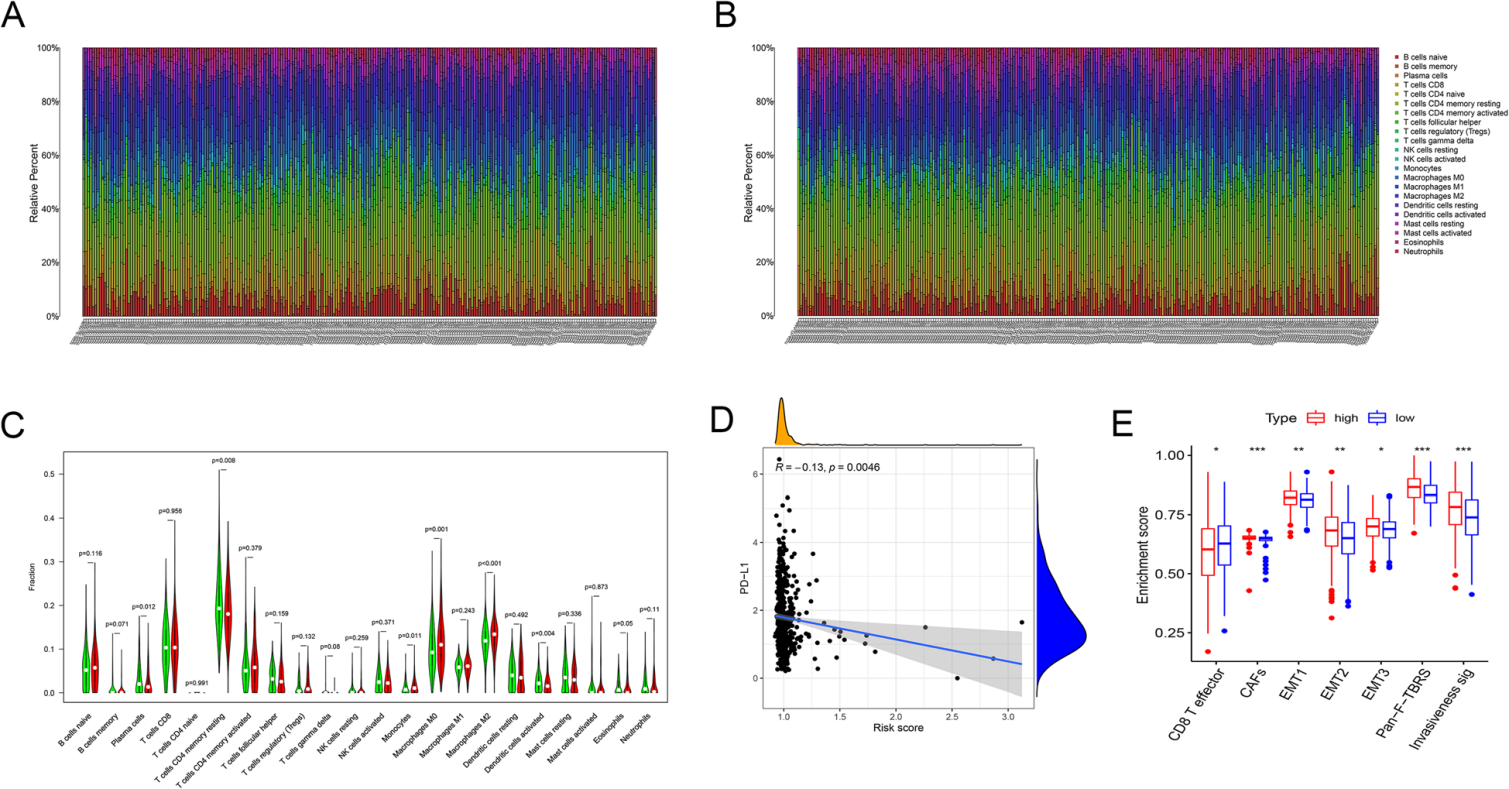
Risk signature was correlated with tumor-infiltrating immune cells. (**A**-**B**) Landscape of immune cell infiltration in the high-risk (**A**) and low-risk (**B**) groups, as determined by the CIBERSORT algorithm. Different color points represent different immune cell types. The vertical axis represents the relative abundance of immune cells; the horizontal axis represents different patients. (**C**) There were significant differences in the abundance of many immune cells between the two groups, especially monocytes (*P* = 0.011), M0 (*P* = 0.001), and M2-like macrophages (*P* < 0.001). Green indicates the low-risk group, and red indicates the high-risk group. (**D**) Risk score was negatively correlated with PD-L1 mRNA (R = − 0.13, *P* = 0.0046). (**E**) The enrichment score of seven cancer invasion-related gene signatures among two subgroups

The differences in abundances of immune cells between the high- and low-risk groups were further investigated. The proportions of 22 immune cells in each sample varied markedly (Fig. 7A and B). When the cut-off value was set to *P* < 0.05, 487 samples of the total set were included in the subsequent analysis. The results showed that monocytes (*P* = 0.011), M0 (*P* = 0.001), and M2-like macrophages (*P* < 0.001) were substantially enriched in the high-risk group, whereas patients in low-risk group had higher levels of plasma cells (*P* = 0.012), resting memory CD4 T cells (*P* = 0.008), and activated dendritic cells (*P* = 0.004) (Fig. 7C). Additionally, we also assessed the correlation between risk score and relative abundances of immune cells. As shown in the correlation heatmap, the results revealed that the risk score was negatively correlated with most of the immune cells (Supplementary Fig. 1A). PD-L1, as an immune checkpoint molecule, is often closely related to immunotherapy response [44, 48]. In this study, the risk score was negatively correlated with PD-L1 mRNA in the training set (R = − 0.13, *P* = 0.0046) (Fig. 7D).

Several gene signatures related to cancer invasion, including invasiveness signature, epithelial-mesenchymal transition (EMT), pan-fibroblast TGFb response signature (Pan-F-TBRS) and two key immune cells in tumor microenvironment (effector CD8+ T and cancer associated fibroblasts (CAFs)) were chosen to further investigation of the role of LMRG score in tumorigenesis and metastasis [49, 50]. The enrichment score of each gene signature was calculated using sample gene set enrichment analysis (ssGSEA) algorithm. Notably, compared with the low-risk group, the high-risk group had a higher enrichment score of EMT- and CAFs-related signatures, while lower enrichment score of CD8+ T effector, which was a critical component for the antitumor immune response (Fig. 7E; Supplementary Fig. 1B-H).

### Mutation landscape of the risk signature in TCGA -CRC cohort

**Fig. 8:**
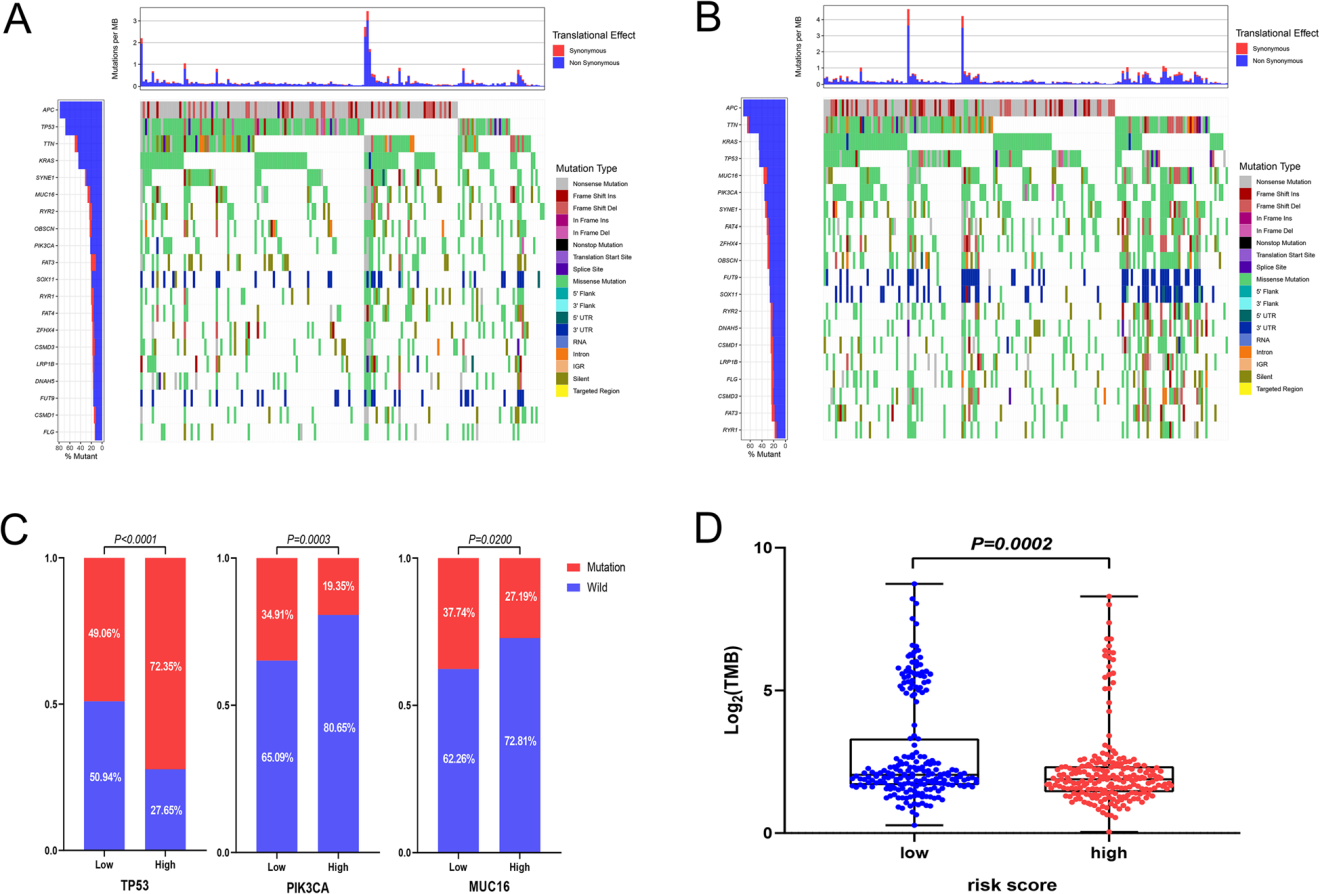
Mutation landscapes of high- and low-risk groups. (**A**-**B**) Waterfall plots showing detailed mutation information for the top 20 most commonly mutated genes in CRC patients in the high-risk (**A**) and low-risk (**B**) groups. Gene names and mutation frequency are shown in the bar chart on the left. Somatic mutation types are indicated by different colors. The bar plot above the diagram shows the translational effects and numbers of mutations per million bases (MB). (**C**) Key genes, including TP53, PIK3CA, and MUC16, showed significant differences in mutation frequency between the high- and low-risk groups. (**D**) The low-risk group had a higher TMB level (*P* = 0.0002)

The top 20 genes with the highest mutation frequency in CRC are shown in the waterfall plots in Fig. 8A and B. The high- and low-risk groups had different genetic mutation landscapes. Genes with crucial biological functions in tumorigenesis, including TP53, PIK3CA, and MUC16, showed significant differences in mutation frequency between the two groups (Fig. 8C). Besides, the top 20 most common mutant genes in CRC, including FAT4, FUT9, LRP1B and ZFHX4, showed significant differences in risk score between mutated- and wild-type group (Supplementary Fig. 2A-D).

A total of 429 samples of TMB data were obtained for this analysis. The results of the analysis showed that the TMB score was higher in the low-risk group than in the high-risk group (*P* = 0.0002, Fig. 8D). We also evaluated the correlation between the risk score and TMB, and found a remarkable correlation between the two variables (r = − 0.19, *P* = 5.7e-05, Supplementary Fig. 2E). This could be one of the reasons for the better prognosis of patients in the low-risk group.

### Distinct biological function pathways characterize high- and low-risk CRC patients in TCGA -CRC cohort

**Fig. 9:**
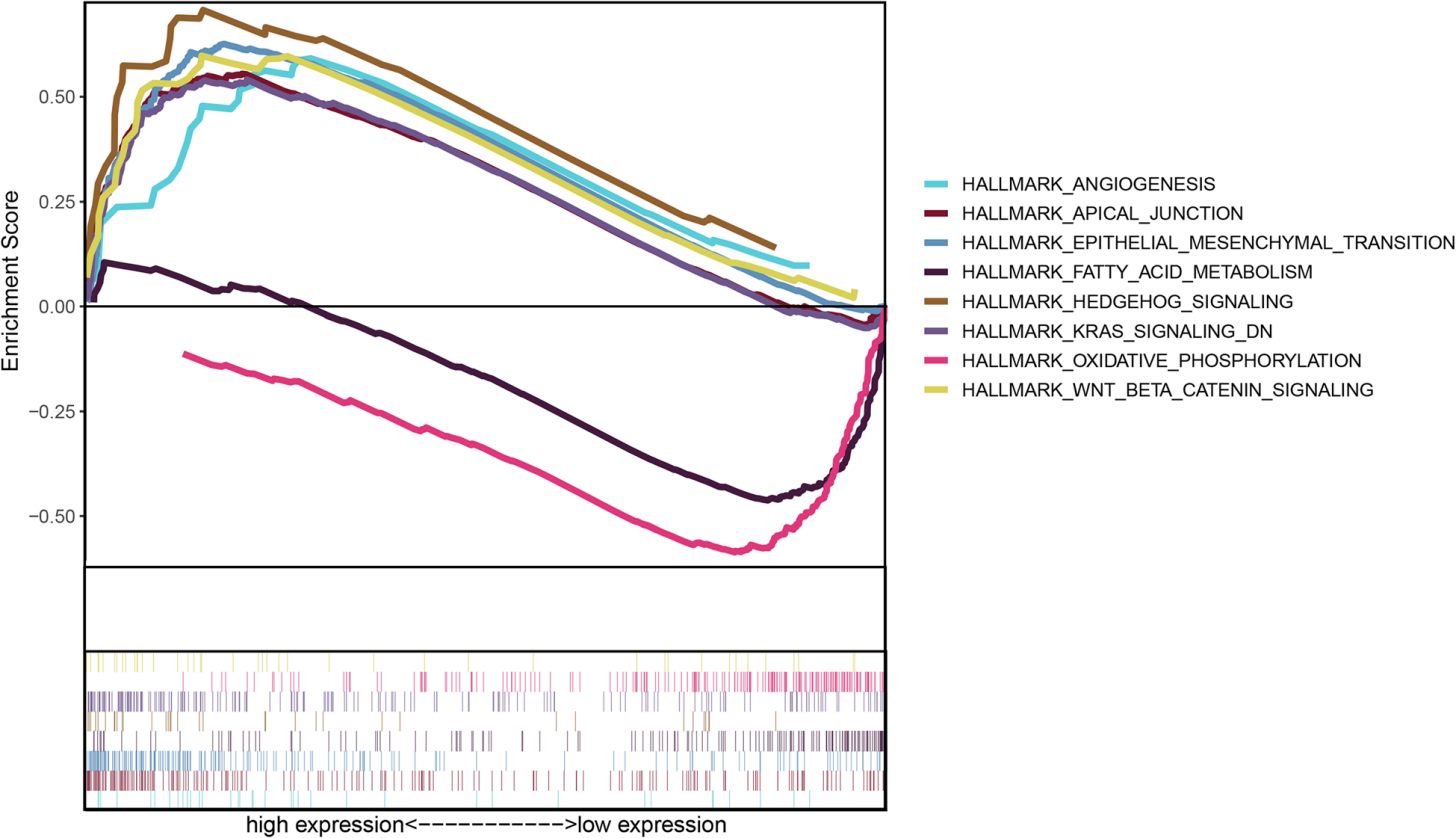
Enriched gene sets in the Hallmark collection for high- and low-risk patients. The high-risk group was associated with Hedgehog signaling, KRAS signaling, Wnt/β catenin signaling, apical junction, epithelial mesenchymal transition, and angiogenesis, whereas functional enrichment in the low-risk group was focused on energy-metabolism-related functions, including fatty acid metabolism and oxidative phosphorylation. Each uniquely colored line represents a particular gene set. The lines above the X axis indicate gene sets enriched in the high-risk group, and the lines under the X axis indicate the gene sets enriched in the low-risk group. The Y axis represents the enrichment score. The higher the absolute values of the score, the more significant the enrichment

GSEA was also performed to explore whether relevant signaling pathways differed between the two risk groups. The results showed that the high-risk group was associated with Hedgehog signaling, KRAS signaling, Wnt/β catenin signaling, apical junction, epithelial-mesenchymal transition, and angiogenesis. Functional enrichment in the low-risk group was focused on energy-metabolism-related functions, including fatty acid metabolism and oxidative phosphorylation (Fig. 9).

## Discussion

In this study, the lipid metabolism statuses of 1023 CRC samples were analyzed using gene expression data from the public databases, and a risk signature (comprising PROCA1, CCKBR, CPT2, and FDFT1) was constructed. This is the first reported CRC prognostic risk signature based on LMRG. Patients with high risk scores had significantly shorter OS than those with low risk scores, in both training and validation sets. The risk signature could well distinguish high-risk CRC patients. The risk signature, as an independent prognostic indicator, might be a beneficial supplement to TNM staging to provide more accurate prognostic information. A nomogram combining the risk signature and clinical characteristics showed better capacity for risk stratification than any clinical parameter alone. Consistent results were obtained in an external cohort from the GEO database (dataset GSE39582). In general, the prediction model based on the four-LMRG risk signature has potential to be an effective and robust prognostic indicator in CRC.

CRC remains the leading cause of cancer-related deaths worldwide, yet it is one of the most preventable cancers. Appropriate screening strategies can reduce the incidence and mortality of CRC by early detection and elimination of precancerous disease. Current CRC screening guidelines are based mostly on two main risk factors: age and family history [51]. However, the fact is that more than 80% of CRC cases occur in individuals with no positive family history in primary relatives [6]. Obviously, the use of these criteria does not fully take into account the heterogeneity of CRC. Lipid metabolic reprogramming frequently emerges in intestinal tumor cells and has been reported to have crucial biological roles in cell proliferation, energy homeostasis, and signal-transduction [52, 53]. Metabolic status is associated with various clinical outcomes in tumor patients, and a metabolism-related risk signature could serve as a prognostic predictor in cancers [54]. Thus, a predictive model that integrates metabolism-related genomic data and clinicopathological characteristics provides hope for primary and secondary prevention of CRC. The value of this signature is to identify high-risk individuals with CRC so that targeted treatment and more stringent postoperative follow-up can be adopted. It could also be used to distinguish low-risk patients to prevent excessive treatment and unnecessary screening.

Another important finding of this work was that lipid metabolism status was significantly correlated with immune infiltration levels in CRC. According to our investigation, the risk signature based on lipid metabolism was related to abundance of monocyte infiltration, especially M2-like macrophages. The high-risk group had higher levels of M2-like macrophages, suggesting that high-risk patients might have a suppressive tumor immune microenvironment (TIME). In this study, patients with low risk score usually have high-level immune cell infiltration, especially CD8+ T effector, exhibiting a “hot” tumor phenotype. In concordance with previous studies, inflammatory phenotype always has better prognosis [55, 56]. Meanwhile, it is found that significant activation of EMT, TGF-β pathway and CAFs in high-risk group, which are hallmarks of stromal activation, showing an immune-excluded phenotype. In immune-excluded tumors, immune cells, especially CD8+ T effector cells, reside in the stroma surrounding tumor cell making direct contact with CAFs rather than tumor cells, resulting in restricting anti-tumor immune response [57, 58]. Furthermore, CAFs-derived exosomes can strikingly shape cancer-promoting microenvironment through involved in multifarious metabolicl processes [59]. It may partly explain significant difference in the clinical outcomes among different metabolic subgroups.

Tumor-associated macrophages (TAMs) are the most important immune cell component of the TIME. Metabolic reprogramming of TAMs shapes their functional subtype [60]. TAMs are often characterized by M2-like macrophages and have a variety of tumor-promoting effects on the tumor microenvironment. Studies have found different metabolic patterns between pro-inflammatory and anti-inflammatory macrophages [61, 62]. Activated pro-inflammatory macrophages often depend on the glycolytic pathway for energy, whereas immunosuppressed macrophages are more inclined to use fatty acid oxidation [63]. Wu et al. found that fatty acids in the TIME, especially unsaturated fatty acids, might promote the polarization of monocytes to M2-like macrophages with a strong immunosuppressive phenotype [64]. Therefore, the lipid-related metabolism risk signature represents alterations in the TIME of CRC. In addition, the risk signature was associated with the immune checkpoint marker PD-L1, suggesting that it has potential as a metabolic marker for immunotherapy in CRC.

Some metabolic regulators have been considered as oncogenes or tumor suppressors. Carnitine palmitoyl transferase II (CPT2) is a rate-limiting enzyme for mitochondrial fatty acid transportation, with a critical role in regulating fatty acid oxidation. Gastric cancer and CRC patients with lower CPT2 expression level have better disease control rates than those with higher CPT2 expression [65]. Blocking fatty acid oxidation by knocking out CPT1A/CPT2 via CRISPR-mediated has been shown to inhibit the invasive phenotype of radiotherapy-resistant breast cancer, suggesting that CPT2 is a potential metabolic target for breast cancer radiotherapy [66]. Besides, CPT2 levels in tumor tissue were found to be associated with oxaliplatin-based chemotherapy sensitivity. Fatty acid catabolism can be inhibited by knocking out CPT2 or using the CPT2 inhibitor perhexiline. This inhibitory effect promotes induction of tumor cell apoptosis by oxaliplatin or other classic chemotherapy drugs [65]. These results shows that CPT2 has the potential to be a metabolic therapeutic target for CRC.

## Conclusions

The results of this study revealed that LMRG have prognostic value in CRC and may be partially involved in the suppressive immune microenvironment formation. LMRG, especially PROCA1, CCKBR, CPT2, and FDFT1, are potential prognostic markers and therapeutic targets for CRC. This lipid metabolism-related prognostic signature represents a potential biomarker for predicting the efficacy of chemotherapy and anti-PD-L1 therapy in CRC.

## Supplementary Information

**Additional file 1: Supplementary Table 1.** Clinical characteristics of the training and validation set.

**Additional file 2: Supplementary Table 2.** The lipid metabolism-related gene sets from the Molecular Signature.

**Additional file 3: Supplementary Fig. 1.** Immune landscape of the risk signature.

**Additional file 4: Supplementary Fig. 2.** Mutation landscape of the risk signature.

## Data Availability

All data can be obtained from TCGA CRC cohort (https://portal.gdc.cancer.gov/repository) and GEO GSE39583 cohort (https://www.ncbi.nlm.nih.gov/geo/).

## Abbreviations

CRC: colorectal cancer
TCGA: the Cancer Genome Atlas
GEO: the Gene Expression Omnibus
LMRG: lipid metabolism-related genes
PCA: the principal components analysis
LASSO: least absolute shrinkage and selection operator
FPKM: fragments per kilobase million
TPM: transcripts per million
ROC: receiver operating characteristic
AUC: area under the curves
TMB: tumor mutation burden
mRNAsi: the mRNA expression-based stemness index
DEGs: differentially expressed genes
OS: overall survival
FDR: false discovery rate
DEGs: differentially expressed genes
HR: hazard ratio
CI: confidence interval
TIME: tumor immune microenvironment
