# Supplementary material for: A lipid metabolism-related genes prognosis biomarker associated with the tumor immune microenvironment in colorectal carcinoma
Source: BMC Cancer. 2021 Nov 5;21:1182. doi: 10.1186/s12885-021-08902-5 (PMC8571885; doi:10.1186/s12885-021-08902-5)
Supplement: Supplementary file 1 — Additional file 1: Supplementary Table 1. Clinical characteristics of the training and validation set. [file 12885_2021_8902_MOESM1_ESM.docx]

**Supplementary Table 1** Clinical characteristics of the training and validation set.

| **Variables** | **Group** | **Training set** | **Validation set** | ***P* value** |
| --- | --- | --- | --- | --- |
|  |  | n=500 | n=523 |  |
| Age |  | 66.07±12.62 | 66.57±13.44 | 0.5350 |
| Gender |  |  |  | >0.999 |
|  | Male | 271 | 284 |  |
|  | Female | 229 | 239 |  |
| Tumor stage |  |  |  | 0.2178 |
|  | I+II | 284 | 277 |  |
|  | III+IV | 216 | 246 |  |
| T Stage |  |  |  | **<0.001** |
|  | T1+T2 | 102 | 54 |  |
|  | T3+T4 | 398 | 469 |  |
| N Stage |  |  |  | 0.4596 |
|  | N0 | 293 | 289 |  |
|  | N1 | 122 | 131 |  |
|  | N2 | 85 | 103 |  |
| M Stage |  |  |  | 0.1353 |
|  | M0 | 427 | 464 |  |
|  | M1 | 73 | 59 |  |
