# Supplementary material for: A lipid metabolism-related genes prognosis biomarker associated with the tumor immune microenvironment in colorectal carcinoma
Source: BMC Cancer. 2021 Nov 5;21:1182. doi: 10.1186/s12885-021-08902-5 (PMC8571885; doi:10.1186/s12885-021-08902-5)
Supplement: Supplementary file 2 — Additional file 2: Supplementary Table 2. The lipid metabolism-related gene sets from the Molecular Signature. [file 12885_2021_8902_MOESM2_ESM.docx]

**Supplementary Table 2**  The lipid metabolism-related gene sets from the Molecular Signature Database.

| Terms | Counts |
| --- | --- |
| GO_PHOSPHOLIPID_METABOLIC_PROCESS | 433 |
| HALLMARK_FATTY_ACID_METABOLISM | 159 |
| KEGG_ GLYCEROPHOSPHOLIPID_METABOLISM | 78 |
| REACTOME_METABOLISM_OF_LIPIDS | 740 |
| REACTOME_PHOSPHOLIPID_METABOLISM | 212 |
