# Supplementary figures and images for: A lipid metabolism-related genes prognosis biomarker associated with the tumor immune microenvironment in colorectal carcinoma
Source: BMC Cancer. 2021 Nov 5;21:1182. doi: 10.1186/s12885-021-08902-5 (PMC8571885; doi:10.1186/s12885-021-08902-5)

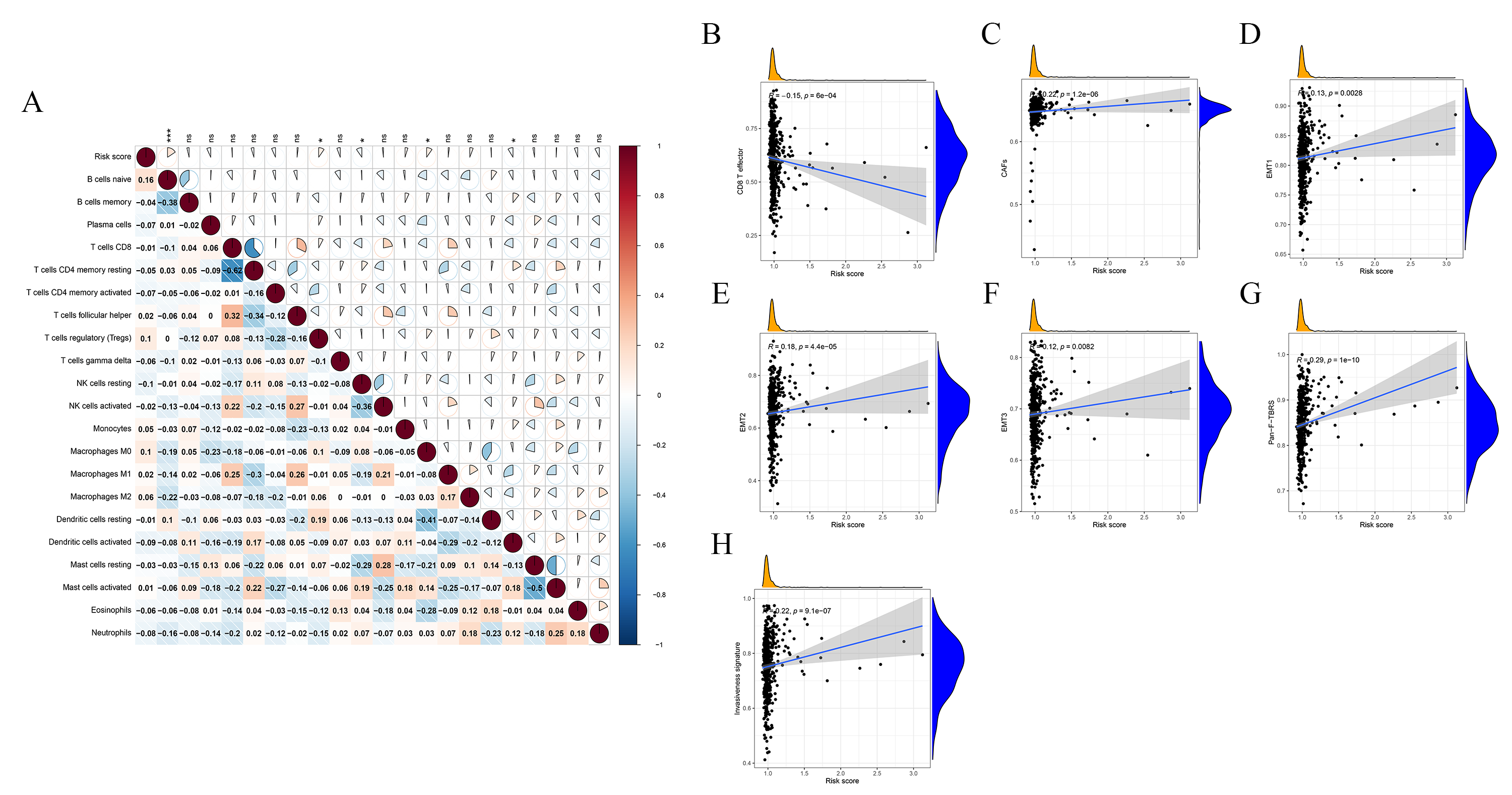

Supplement: Supplementary file 3 — Additional file 3: Supplementary Fig. 1. Immune landscape of the risk signature. [file 12885_2021_8902_MOESM3_ESM.tif]

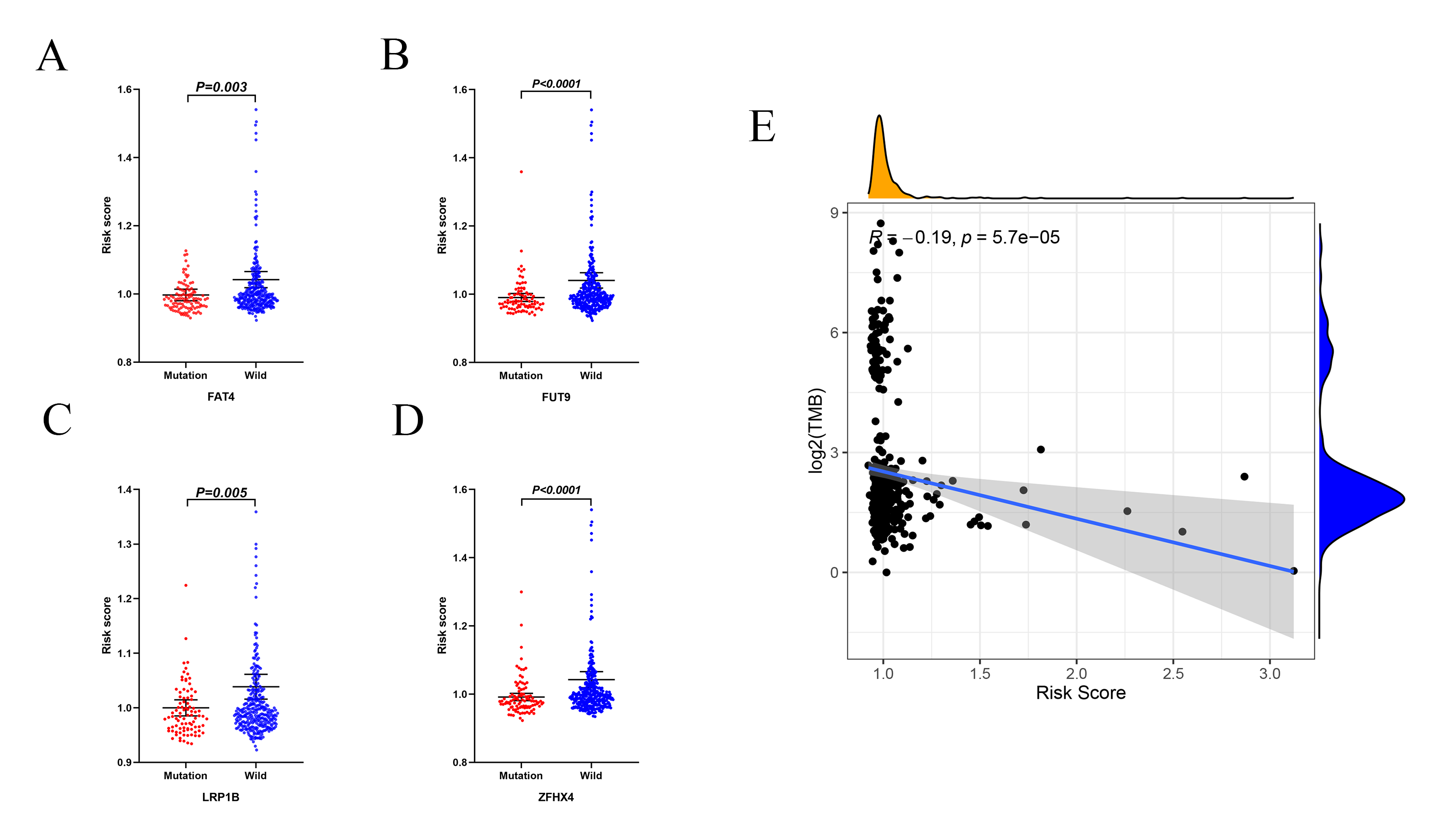

Supplement: Supplementary file 4 — Additional file 4: Supplementary Fig. 2. Mutation landscape of the risk signature. [file 12885_2021_8902_MOESM4_ESM.tif]
